# Methods for mapping and categorization of DNA sequence reads from allopolyploid organisms

**DOI:** 10.1186/1471-2156-16-S2-S4

**Published:** 2015-04-23

**Authors:** Justin T Page, Joshua A Udall

**Affiliations:** 1 Department of Biology, Brigham Young University, Provo, UT 84601, USA; 2 Department of Plant and Wildlife Sciences, Brigham Young University, Provo, UT 84601, USA

**Keywords:** PolyDog, homoeolog, homeolog, allopolyploid, read categorization

## Abstract

Genome read categorization determines the genome of origin for sequence reads from an allopolyploid organism. Different techniques have been used to perform read categorization, mostly based on homoeo-SNPs identified between extant diploid relatives of allopolyploids. We present a novel technique for read categorization implemented by the software PolyDog. We demonstrate its accuracy and improved categorization relative to other methods. We discuss the situations in which one method or another might be most appropriate.

## Background

Allpolyploid organisms are a type of polyploid in which two or more genomes from different ancestor species are brought together in a single nucleus. This genome doubling has radical effects on the genome. It causes immediate changes, termed "genomic shock", that affect the genetic and epigenetic state of the genome. In the long term, the genome doubling alters the course of evolution as two originally independent and self-sufficient genomes interact and develop together.

Allopolyploids are economically important to human society because there are many allopolyploid crops, including cotton, peanut, soybean, and Brassica. Analysis of these allopolyploids is complicated by the presence of multiple genomes. For example, single nucleotide polymorphisms (SNPs) that distinguish the co-resident genomes (homoeo-SNPs) can be confounded with SNPs that segregate in a Mendelian fashion (allele-SNPs).

Genome read categorization is the process of assigning DNA or RNA sequence reads from an allopolyploid organism to their singular genome of origin. Separating the genomes of an allopolyploid empowers researchers to identify true allele-SNPs and compare the parallel evolution of duplicated genes.

Common approaches to genome read categorization often involve the use of a single reference genome, belonging to a single diploid relative of one of the genomes from the allopolyploid, even if both diploid genome sequences are available. Sequence reads from diploid relatives of all constituent genomes are mapped to this reference, then SNPs distinguishing the diploid relatives are inferred to represent homoeo-SNPs that would distinguish the genomes of the allopolyploid [1]. Reads from the allopolyploid can then be categorized to their genome of origin based on how closely it matches the haplotypes of the two parents. Note that, while a whole-genome reference sequence is desirable, the same strategy can be used with draft and/or transcriptome assemblies as a reference sequence. We previously developed PolyCat, which uses this approach [2]. PolyCat considers homoeo-SNPs overlapped by each mapped read, and counts the bases at SNP locus to assign genome of origin for the read. If a threshold majority (default 75%) of counts match one of the genomes, the read is categorized to that genome. Multiple SNPs overlapped by a single read are evaluated for consistency of the genome assignment. Other tools have been developed using similar approaches to this problem, including HANDS and SNiPloid [3,4].

Along with read categorization, a researcher should consider a few issues when analyzing sequence data from an allopolyploid. First, if diploid A is used as the reference sequence, there will likely be an inherent mapping bias favoring reads from the A_T _genome of the tetraploid over the B_T _genome of the tetraploid (where the 'T' subscript distinguishes between the respective genomes in tetraploid nucleus). This can be alleviated through the use of GSNAP's SNP-tolerant mapping, which can take an index of known homoeo-SNPs identified between the diploid relatives and allow specified mismatches at those positions without penalizing the sequence alignment [5]. Second, even when the mapping bias between diploids is accounted for, there may also be differences in the genetic distances between the tetraploid genomes and their respective diploid relatives. For example, the A genome could be better approximation of the A_T _genome than the B genome is for the B_T _genome. If a static SNP index is being used, iterative development of SNP-indices may alleviate this problem by categorizing reads from a tetraploid then calling SNPs between the resulting genomes to generate a set of homoeo-SNPs that more closely represents the state of the tetraploid, rather than of the diploids. Finally, read categorization based on SNPs is limited by the ability of reads from one genome to map to the reference sequence of another genome. Wherever reads can map, homoeo-SNPs can potentially be identified. However, read categorization will only work if polymorphisms also exist at those loci.

Cotton species provide an excellent framework for the study of allopolyploidy and the development of specialized software. Allotetraploid cotton, which accounts for over 90% of cotton production worldwide, is the result of a hybridization and polyploidization event that occurred 1-2 million years ago (mya). At least 5 allotetraploid species arose from this single polyploidization. The parents of this event were A-genome and D-genome diploids. The A-genome diploids *Gossypium herbaceum *(A_1_) and *G. arboreum *(A_2_) are the closest extant diploid relatives of the allotetraploid A-genome (A_T_), while *G. raimondii *(D_5_) is the closest extant diploid relative of the allotetraploid D-genome (D_T_). The A- and D-genomes diverged ~10 mya and both have 13 chromosomes. The A-genome is about twice the size of the D-genome (1.7 Gbp vs 0.9 Gbp), but the two genomes are largely collinear. The difference in length is largely made up of transposable elements. Allotetraploid cotton is a good model for research on polyploid genomes because the genome is relatively static and close diploid relatives are known for the genomes of the allotetraploid.

**Figure 1:**
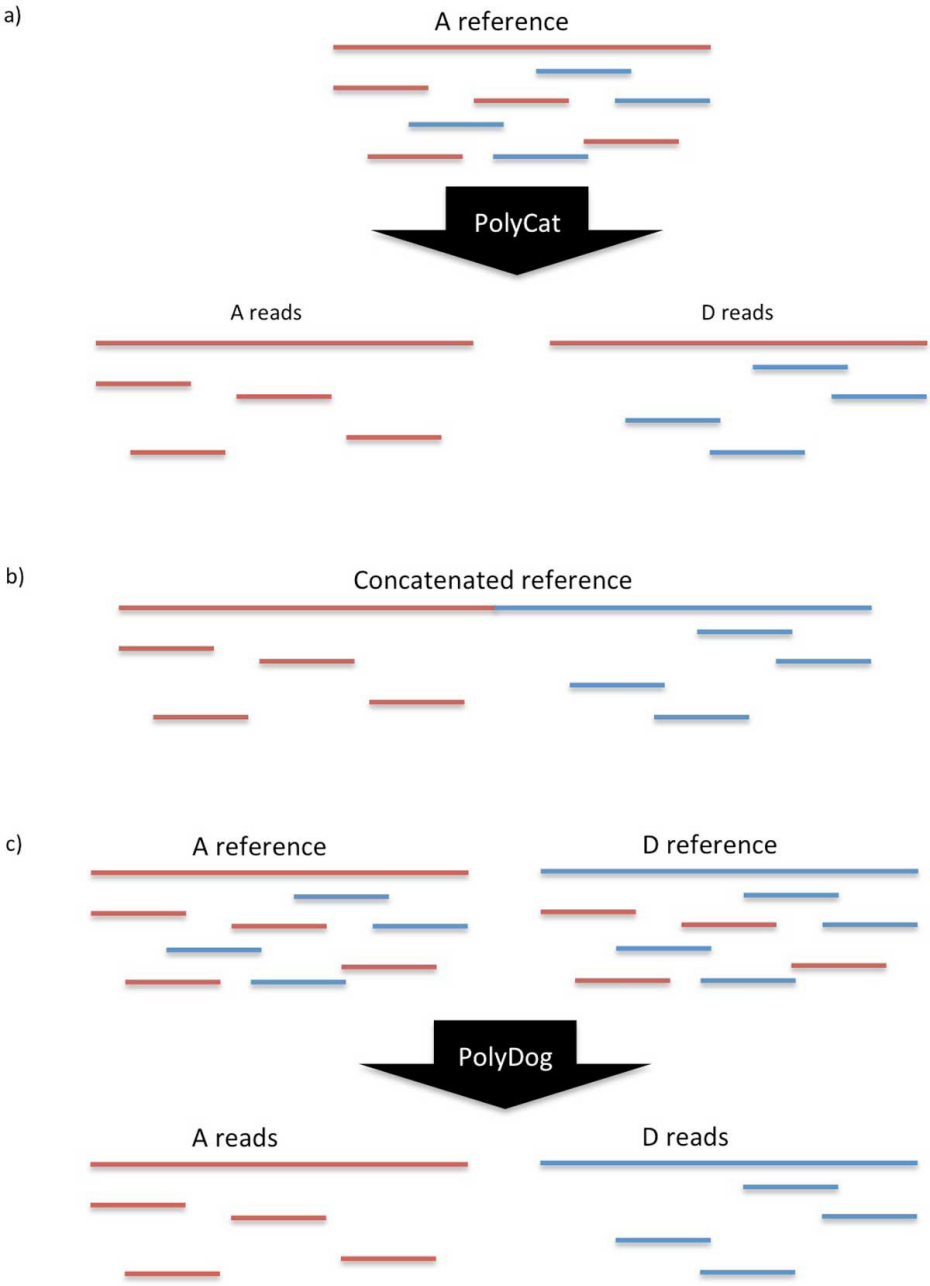
**Methods for read categorization**. In all images, red lines represent the A-genome while blue lines represent the D-genome. Idealized forms of these methods are shown, ignoring structural differences, perfect conservation, and other sources of complication and error. With PolyCat, (a) homoeo-SNPs are first identified between the consensus sequences for already known A- and D-genome reads. Then all reads are mapped to a single reference sequence (A-genome in the example) and PolyCat categorizes them by source genome. With the full reference method (b), reads are mapped to a concatenation of the A- and D-genome reference sequences, so reads will naturally map to the part of the reference that represents its appropriate genome. With PolyDog (c), the same set of reads from an allotetraploid is mapped to both the A- and D-genome references. Then PolyDog examines each pair of mappings and categorizes that read to its genome of origin.

Here we present a new approach to read categorization that simultaneously uses data from two reference sequences, one for each genome of an allotetraploid. This dual-reference approach is implemented by our software called PolyDog. We compared the effectiveness of the dual-reference method to the results of read categorization using either reference alone. We also compared the dual-reference method of mapping to a concatenation of two genome references, rather than to just one or the other (Figure 1.).

PolyDog, along with PolyCat, is available for open source download as part of the BamBam package (https://sourceforge.net/projects/bambam/).

## Results

### PolyDog implementation

PolyDog processes two alignments (in BAM format) at once. These BAM files are made with the same set of reads, but are mapped to different references: in this case, the A_2 _reference and the D_5 _diploid reference, related to the A_T _and D_T _genomes of allotetraploid cotton. PolyDog examines each read on the basis of its mappings to both references and decides which reference the read matches more closely. Each read is analyzed based on 4 criteria:

1. Whether the read mapped

2. What mapping quality score (MAPQ) it had

3. How long the alignment was

4. How many bases matched the reference exactly (insertions and deletions are penalized as a mismatch)

**Figure 2:**
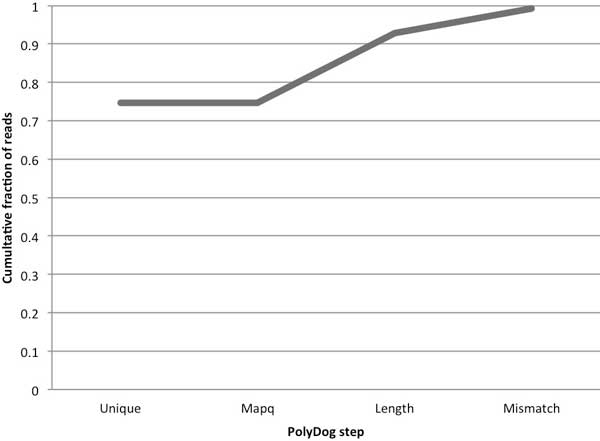
**Categorization by each PolyDog step**. Reads were first categorized by PolyDog based on unique mapping to one genome or another, then based on MAPQ, alignment length, and number of mismatches. In our tests, GSNAP did not calculate different MAPQ scores for each alignment, so MAPQ was not helpful in categorization. Fractions shown are relative to the total number of mapped reads, and are averaged over 3 allotetraploid datasets.

These factors are factored serially, so the quality scores of the alignments are only considered if the read mapped to 1 or more locations; the alignment length is only considered if the read mapped in both references with equal MAPQ score, *etc*. If one mapping scores better than the other in a criterion, the read is categorized to the genome corresponding to the better mapping. In the tetraploid tests discussed below, nearly 75% of reads were categorized based on unique mapping to one genome or the other (Figure 2). Differences in the length of reads aligned to each reference accounted for another 18%. Less than 1% of reads mapped to at least one reference but could not be categorized by any method. The relative contribution of each step will likely vary greatly based on the distance and nature of the relationship between the reference genome sequences.

When running PolyDog, reads are reported as belonging to the A-genome, D-genome, or unknown N-genome. These N reads are made up primarily of reads that map equally well to both reference sequences.

### Comparative analysis

Whole-genome shotgun reads were used to compare the different mapping and categorization methods. All reads were 100 bp paired-end Illumina reads.

Reads were mapped to 2 genome references. The 13 chromosomes of *G. arboreum *represented the A-genome, while the 13 chromosomes of *G. raimondii *represented the D-genome [6,7]. Three allotetraploid species were used to test real application: *G. tomentosum *(AD_3_), *G. darwinii *(AD_4_) and *G. mustelinum *(AD_5_). They each have 26 chromosomes (2n = 4x = 52), 13 from an A-genome ancestor and 13 from a D-genome ancestor. Mappings were performed using GSNAP [5]. Only unique best mappings were accepted ("-n 1 -Q"). For PolyCat (but not for PolyDog or the full reference method), SNP-tolerant mapping was used ("-v" option) with the same set of homoeo-SNPs later used for read categorization by PolyCat.

PolyDog was run with paired-end support turned on, allowing fragments to be categorized as a single unit. Reads that mapped equally well to both references were rejected.

PolyCat was also run with paired-end support. A minimum vote majority of 75% per fragment was used. The SNP-index used for categorization was specific to each of the tetraploids. Initially, reads were mapped and categorized using a SNP-index based on homoeo-SNPs inferred from alignments of 6 A-genome and 4 D-genome diploids. Then SNPs were identified between allotetraploid reads categorized as A-genome and allotetraploid reads categorized as D-genome. Those SNPs were identified for each allotetraploid (AD_3_, AD_4_, and AD_5_) and used for (re-) mapping and categorization in these tests.

For the full reference method, reads were "categorized" based on the reference chromosome they mapped to.

### Error analysis

Three diploids were used to test the accuracy of genome categorization by different methods: *G. herbaceum *(A_1_-97), *G. arboreum *(A_2_-34), and *G. raimondii *(D_5_-2). These reads were treated in the same manner as the tetraploid reads: mapping with GSNAP followed by categorization by PolyCat and PolyDog. For the PolyCat tests, a SNP-index was used, based on homoeo-SNPs identified between 6 A-genome diploids and 4 D-genome diploids.

Categorizing diploid reads should be redundant because the genome of origin is already known for each read. But categorized diploid reads can be used as a useful measure for the accuracy of categorization methods, as every read from the D-genome diploid SHOULD be categorized as belonging to the D-genome. As such, the fraction of mapped reads that categorize to the A-genome instead of the D-genome approximates the error rate of that categorization method. Using an A-genome diploid, the fraction of mapped reads that categorize to the D-genome instead of the A-genome approximates the error rate. Note that this system for measuring error rates only works because each read pair is mapped and categorized independently by all the methods analyzed in this study.

**Figure 3:**
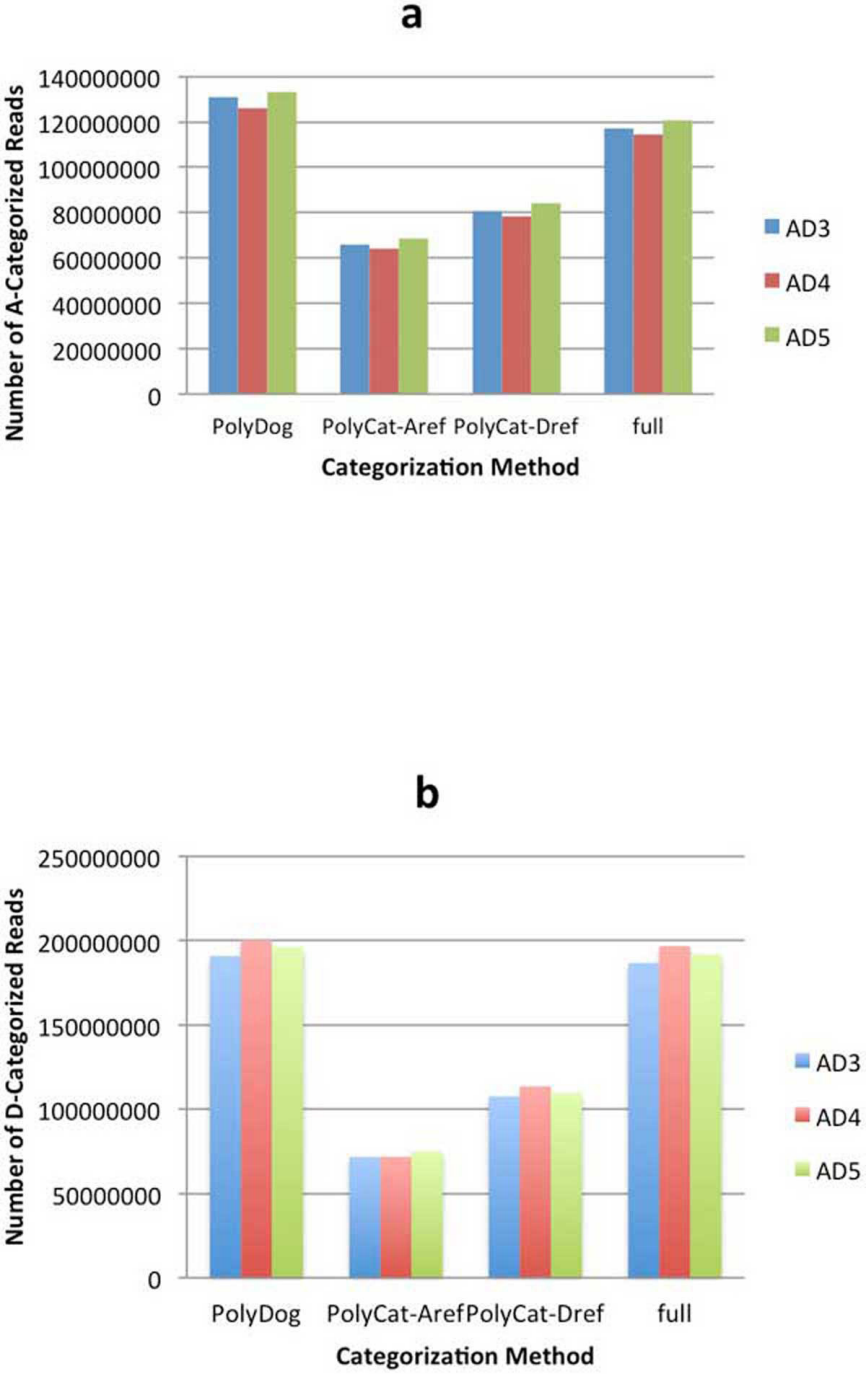
**Categorization results by method**. Number of reads categorized to the A-genome (a) and to the D-genome (b). Reads from three allotetraploid cotton species--AD3(blue), AD4(red), and AD5(green)--were categorized by PolyDog, PolyCat using the A-genome as reference, PolyCat using the D-genome as reference, and the full reference method.

PolyDog was able to categorize slightly more reads than the full reference method, and both of these methods categorized far more reads than the PolyCat method, regardless of whether the A-genome or D-genome reference was used (Figure 3). The disadvantage of PolyCat is that it can only categorize reads in the homoeologous regions of the genome. The A-genome has hundreds of megabases of sequence that are not present in the D-genome, and even the smaller D-genome also has many regions that are absent in the A-genome. But PolyCat can only categorize reads where homoeo-SNPs are identified, and homoeo-SNPs can only be identified if the same region exists in both genomes.

PolyDog slightly outperformed the full reference method. This was largely because unique best mappings (GSNAP options -n 1 -Q) were required in the initial reference mapping. So a read that mapped equally well to the A-genome and D-genome versions of a locus would be unmapped in the full reference method. With PolyDog, however, the read would be mapped in both of the separate mappings. When PolyDog examines such mappings, it is able to investigate the difference between them with a finer resolution than GSNAP did when looking for the mapping. As a result, it may be able to assign the read to one genome. The ability of PolyDog to do this depends on the confidence thresholds used by the mapper and by PolyDog. But in general, PolyDog is and can be more aggressive than GSNAP in choosing a best mapping for a read because it is aware of the specific relationship between the two proposed mappings as pertaining to homoeologous loci.

**Figure 4:**
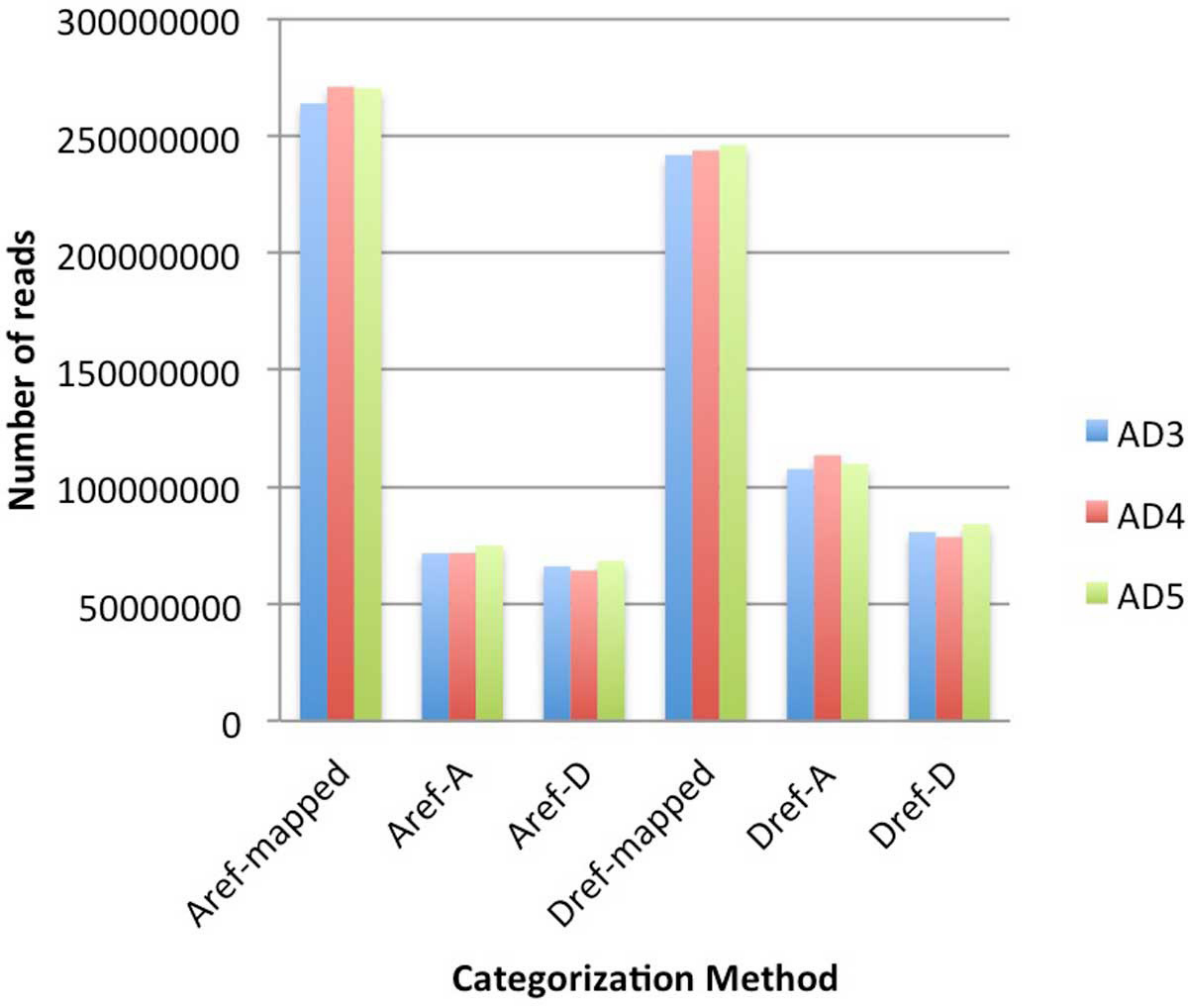
**PolyCat performance**. Reads from three allotetraploid cotton species--AD3(blue), AD4(red), and AD5(green)--were mapped by GSNAP with SNP-tolerant mapping and categorized by PolyCat using either the A-genome or D-genome reference sequence. The total number of mapped reads in each case is shown (Aref-mapped and Dref-mapped), as well as the number of reads categorized to the A (Aref-A and Dref-A) and D genomes (Aref-D and Dref-D).

With the PolyCat tests, more reads were mapped to the A-genome reference sequence than to the D-genome reference sequence (69.4% vs. 63.2%), but less reads were categorized (Figure 4). The increased mapping rate is likely due to the large amount of non-homoeologous sequence in the A-genome. Allotetraploid reads from a non-homoeologous region can map to the A-genome but not the D-genome, thus increasing the mapping rate for the A-genome reference. However, the homoeologous portion of the genome is biologically the same size in both genomes, so it should be the same size in both references. But more homoeo-SNPs have been identified in the D-genome reference (28 M) than in the A-genome reference (15 M). As a result, PolyCat can analyze reads mapped to the D-genome reference with greater resolution. Thus, categorization rates were lower with an A-genome reference sequence.

**Figure 5:**
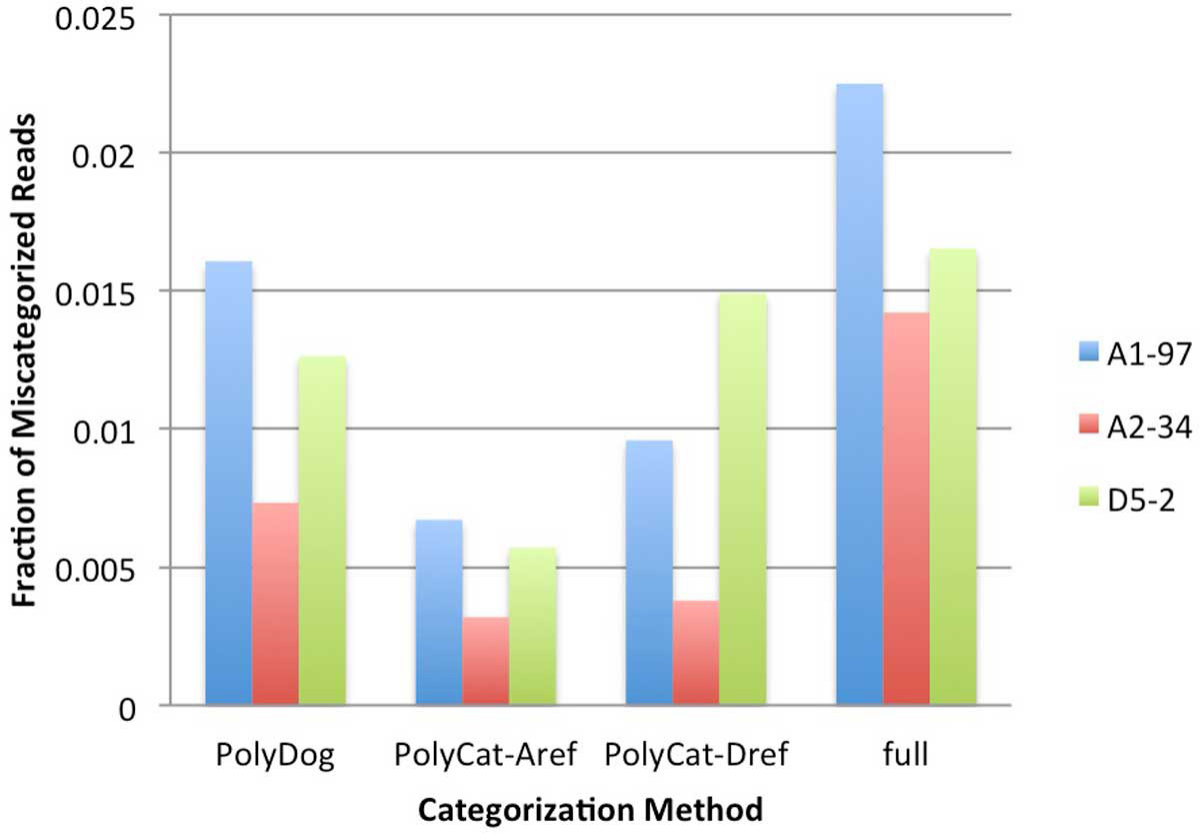
**Error rates in categorization**. Three diploid cotton species--A1-97 (blue), A2-34 (red), and D5-2 (green)--were categorized by PolyDog, PolyCat using the A-genome as reference, PolyCat using the D-genome as reference, and the full reference method. The error rate shown is the number of reads categorized to the wrong genome divided by the number of mapped reads.

PolyDog and the full reference method had higher error rates than the PolyCat methods (Figure 5.). PolyCat is much more conservative, only using high confidence homoeo-SNPs and focusing on regions that can easily be distinguished by genome. Consequently, PolyCat categorizes far fewer reads but with a correspondingly low error rate. With the A-genome reference, PolyCat has less homoeo-SNPs to work with and thus categorizes even less reads with a correspondingly low error rate. Between PolyDog and the full reference method, PolyDog had a slightly lower error rate, likely for the same reason as it had a slightly higher categorization rate. The highest error rate of any method was less than 2.5% and most error rates were about 1%, suggesting that all these methods can be used to provide highly confident results (~99%).

In PolyDog and the full reference method, the highest error rate was observed in A_1_-97 because the other two species (A_2_-34 and D_5_-2) were each represented in one of the reference genome sequences. In PolyCat, the homoeo-SNPs were based on diploids from all three species, so the A_1_-97 did not have as much of a higher error rate. A_2_-34 consistently exhibited a much lower error rate than either of the other species. The reason for this superior accuracy with A_2_-34 is unclear.

The quality and completeness of the reference sequences can have a massive effect on error rate. This is readily observable in PolyDog output. PolyDog's error can be reported as the number of reads from a D-genome diploid that mapped to the D-genome reference but were ultimately (erroneously) categorized as A-genome reads. If you instead consider the number of D-genome reads that mapped to the A-genome reference and were (erroneously) categorized as A-genome reads, the number will likely be higher than with the previous measurement. This is because the reference being used is the wrong categorization type, so it's easier for reads mapped to that reference to look like the wrong categorization type. With D_5_-2 reads, this increase of error as observed using a different reference sequence is about 2x (3.66 M -> 7.20 M reads). With A_1_-97 and A_2_-34 reads, this increase is 17x (4.04 M -> 141.25 M reads) and 35x (7.45 M -> 127.72 M reads), respectively. A likely cause for this asymmetry is the relative completeness and quality of the A- and D-genome reference sequences. This effect will vary greatly depending on the relative completeness of the reference sequences used, as well as the distance between the diploid relative and the allotetraploid being analyzed.

## Conclusions

Using both reference sequences, either through PolyDog or the full reference method, is beneficial because it allows analysis of both the homoeologous and non-homoeologous portions of the genomes. However, there are still reasons to use a single reference sequence, such as with PolyCat. First, a reference sequence may only be available for one of the genomes in an allopolyploid, or the reference sequence of one genome may be largely incomplete. Second, if a SNP-index is used to properly alleviate mapping biases and categorize reads, a single reference sequence facilitates a comparison of homoeologs with each other. This can aid in the identification of allele-SNPs and other Mendelian polymorphisms. It also facilitates direct quantitative comparison, as for gene expression analysis. In contrast, PolyDog can categorize reads in regions that are unique to one genome or the other. This may introduce a bias in the analysis. PolyDog would be better suited to qualitative analyses, such as genotyping loci and building phylogenetic trees, because it can use reads from the unique parts of the genome.

Perfectly conserved regions cannot be analyzed by read categorization because no difference in sequence identity can be exploited. Highly repetitive regions are also likely to be uncategorizable. It is possible that a region that is highly conserved between diploid species may have diverged in the polyploidy. The genome shock associated with polyploidization may cause almost immediate changes in the polyploidy. Or the presence of two copies of each gene in the same nucleus may result in divergent gene fates: neo-functionalization, sub-functionalization, or non-functionalization [8]. Regardless, PolyCat may detect these polymorphisms because a read may stretch from a categorizable region into a non-categorizable region. Longer reads make this more likely. In addition, using paired-end data ("-p" option) allows a whole fragment to be categorized together, thereby reaching even further into an otherwise uncategorizable region. If SNPs are identified in this manner, a new index based on the allotetraploid itself may be constructed, facilitating further analysis. In addition, such an allotetraploid-specific SNP index has the benefit of not including homoeo-SNPs that resulted from autapomorphies in one of the diploid relatives.

A SNP-index is not used by PolyDog, and it is recommended that SNP-tolerant mapping not be used in preparing BAM files for analysis by PolyDog. This is because PolyCat and PolyDog act on fundamentally opposite principles in the mapping stage. PolyCat seeks to map reads from the "wrong" genome to a reference sequence (*e.g*., map A-genome reads to a D-genome reference). Then it categorizes the reads to sort out genomic identity. On the other hand, PolyDog seeks to NOT map reads from the "wrong" genome to a reference sequence. It is desirable (with PolyDog) that a read from the A-genome not map to the D-genome. Then PolyDog may easily recognize the genomic origin of the read.

PolyDog's advantage over the full reference method is that PolyDog can leverage the knowledge of the homoeologous relationship between loci in different genomes and distinguish it from the possible paralogous relationship between loci within a genome. In effect, PolyDog allows multiple hits when those multiple hits are on different genomes but disallows multiple hits when they're on the same genome. PolyDog does this by applying different standards to distinguish homoeologs from those used to distinguish paralogs. Stricter settings and larger margins are needed to confidently avoid paralogous mapping, but looser settings and minimal margins can be used to decide to which homoeolog a read belongs. PolyDog can require unique best mapping without having to discard reads that map comparably well to both genomes. In contrast, the full reference method must either 1) allow multiple hits for each read or 2) require unique best mapping. Option 1 allows a read to map to both genomes, but it also allows a read to map to multiple loci within one genome, which is often undesirable. Option 2 avoids this, but it also throws away some reads that map to homoeolgous loci. PolyDog takes the benefits of both. It maps each read to just a single locus in each genome separately. Then PolyDog analyzes and compares those best mappings from each genome. Thus, a read can map to two loci, but only if they're on different genomes.

While PolyDog performs better than the full mapping method, the difference is small. Because of this, another consideration becomes important in deciding which method to use for a specific experiment. PolyDog ultimately results in reference mappings made to each of the reference genome sequences. For each reference, there is a single mapping for each genome. Thus for cotton, PolyDog produces a BAM file of A-genome reads mapped the A-genome reference, A-genome reads mapped to the D-genome reference, D-genome reads mapped to the A-genome reference, and D-genome reads mapped to the D-genome reference. These results can be very useful for comparisons where each genome should be considered separately [9]. They can also be useful for identification of genetic markers such as SNPs [10]. In contrast, the full reference method results in a single mapping of all reads against a concatenated reference. Such an output may be more appropriate for comparisons of species, where the character of the distinct genomes is not of interest.

When reference genome sequences are available for relatives of each genome of an allopolyploid, read categorization with PolyDog can leverage both sequences to maximize read categorizability with high (~99%) confidence. When dealing with unique best mappings for each read, PolyDog is a better option than simply mapping to a concatenation of the reference sequences, with higher categorization rates and lower error rates.

When a reference genome sequence is only available for the relative of one genome from the allopolyploid, read categorization based on homoeo-SNPs, whether through PolyCat, SNiPloid, HANDS, or some other tool, is an excellent and high confidence solution. However, analysis will be limited to regions that are present in both genomes, limiting the analysis of copy number variants.

Even if reference genome sequences are available for all genomes of the allopolyploid, it may still be preferable to use a single reference genome sequence followed by a tool like PolyCat. This will serve to minimize mapping and categorization biases between the genomes, facilitating quantitative analyses such as gene expression studies.

## Competing interests

The authors declare that they have no competing interests.

## Authors' contributions

JP wrote and tested the software and prepared the manuscript. JU is JP's advisor, provided the data and resources for development and testing, and edited the manuscript. JP and JU approved the final manuscript.

## Declarations

The publication costs for this article were funded by Cotton Inc. and the National Science Foundations' Plant Genome Research Program.

This article has been published as part of *BMC Genetics *Volume 16 Supplement 2, 2015: Selected articles from The 11th Annual Biotechnology and Bioinformatics Symposium (BIOT-2014): Genetics. The full contents of the supplement are available online at http://www.biomedcentral.com/bmcgenet/supplements/16/S2.
